# A case of hemichorea in *RNF213*-related vasculopathy

**DOI:** 10.1186/s12883-021-02061-7

**Published:** 2021-01-22

**Authors:** Satoshi Hosoki, Takeshi Yoshimoto, Masafumi Ihara

**Affiliations:** Department of Neurology, National Cerebral and Cardiovascular Center, 6-1 Kishibe-Shimmachi, Suita, Osaka, 564-8565 Japan

**Keywords:** Carotid stenosis, Chorea, *RNF213*, Moyamoya disease, East-west gradient

## Abstract

**Background:**

Internal carotid artery (ICA) stenosis has been recently reported to cause hemichorea, mainly in East Asia. The East Asian-specific p.R4810K variant of *RNF213*, a susceptibility gene for moyamoya disease (MMD), accounts for up to 25% of sporadic ischemic stroke with ICA stenosis cases in East Asia. However, as *RNF213*-related vasculopathy does not meet the diagnostic criteria for MMD, the creation of a new disease category has been suggested. Here, we report the first case of hemichorea in *RNF213*-related vasculopathy.

**Case presentation:**

An 81-year-old woman was admitted to our hospital with choreic movements in the periphery of the right extremities at rest. Though head magnetic resonance imaging showed no fresh or old cerebral infarction, ^123^I-iodoamphetamine-single photon emission computed tomography showed cerebral blood flow of < 80% in the anterior territory of the left middle cerebral artery (MCA) in a resting state and cerebrovascular reactivity of < 10% in the broader area supplied by the left MCA after acetazolamide challenge. Head magnetic resonance angiography and digital subtraction angiography revealed left ICA C1 portion stenosis with compromised collateral vessels. Involuntary movements resolved with haloperidol administration within 3 days, without apparent recurrence from continuation of the medication for a year. Genetic testing revealed the presence of the heterozygous *RNF213* p.R4810K variant.

**Conclusions:**

Chorea is thought to be caused by damage to circuitry connecting the basal ganglia with the cerebral cortex, as found in cases of MMD, which possess aberrant vessels in the basal ganglia. However, aberrant vessels and cerebral infarctions were not observed in the basal ganglia in the current case, decreasing the likelihood of a role in chorea. Alternatively, as RNF213 regulates vascular endothelial function and angiogenesis, dysregulation may impair the neurovascular unit and damage basal ganglia circuitry, contributing to the development of chorea. This case may renew interest in the concept of *RNF213*-related vasculopathy and the pathophysiological mechanisms behind chorea in ICA stenosis.

## Background

Chorea is characterized by abrupt involuntary movements resulting from a continuous flow of random muscle contractions [1]. Internal carotid artery (ICA) stenosis rarely causes chorea but has been recently reported to cause hemichorea. These reports emanate mainly from East Asia [2–11] (Table 1), though the reason for the regional difference is not clear. Nonetheless, chorea is found relatively frequently in 3.3–4.2% of patients with ICA stenosis caused by rare moyamoya disease (MMD) [12]. MMD shows a characteristic ‘East-West gradient’ geographical pattern of the disease prevalence because of the East Asian-specific *RNF213* p.R4810K variant [13]. Furthermore, this nonsynonymous p.R4810K variant accounts for up to 25% of sporadic ischemic stroke with ICA stenosis in East Asia, even without meeting the diagnostic criteria of MMD [14], which has led to a novel disease concept of *RNF213*-related vasculopathy [13]. Here, we report the first case of hemichorea in a *RNF213*-related vasculopathy other than MMD, which may help define the clinical spectrum of *RNF213*-related vasculopathy.

**Table 1:** Cases of hemichorea caused by internal carotid artery stenosis, which did not meet the diagnostic criteria of moyamoya disease

Age	Sex	Side of chorea	Basal ganglia infarction (if not, location of infarction)	Country	Reference
77	Female	Rt.	Bil. basal ganglia, rt. caudate nucleus and bil. centrum semiovale	Japan	[2]
60	Male	Lt.	No basal ganglia infarction (Rt. centrum semiovale)	England	[3]
75	Male	Rt.	No basal ganglia infarction (Lt. posterior parietal gray matter)	England	[4]
73	Male	Rt.	No basal ganglia infarction (Lt. anterior parietal lobe)	England	[4]
75	Male	Lt.	No basal ganglia infarction (Rt. fronto-parietal subcortical white matter and centrum semiovale)	Japan	[5]
75	Male	Lt.	No basal ganglia infarction (Bil. corona radiata)	Japan	[2]
67	Female	Lt.	No basal ganglia infarction (Rt. anterior border zone)	Korea	[6]
72	Female	Rt.	No infarction	England	[4]
81	Male	Rt.	No infarction	Spain	[7]
72	Male	Bil.	No infarction	Japan	[8]
73	Male	Lt.	No infarction	Japan	[9]
73	Male	Lt.	No infarction	Japan	[10]
71	Male	Lt.	No infarction	Japan	[11]
81	Female	Lt.	No infarction	Japan	This case

Abbreviations: *bil* Bilateral, *rt* Right, *lt* Left

## Case presentation

**Fig. 1:**
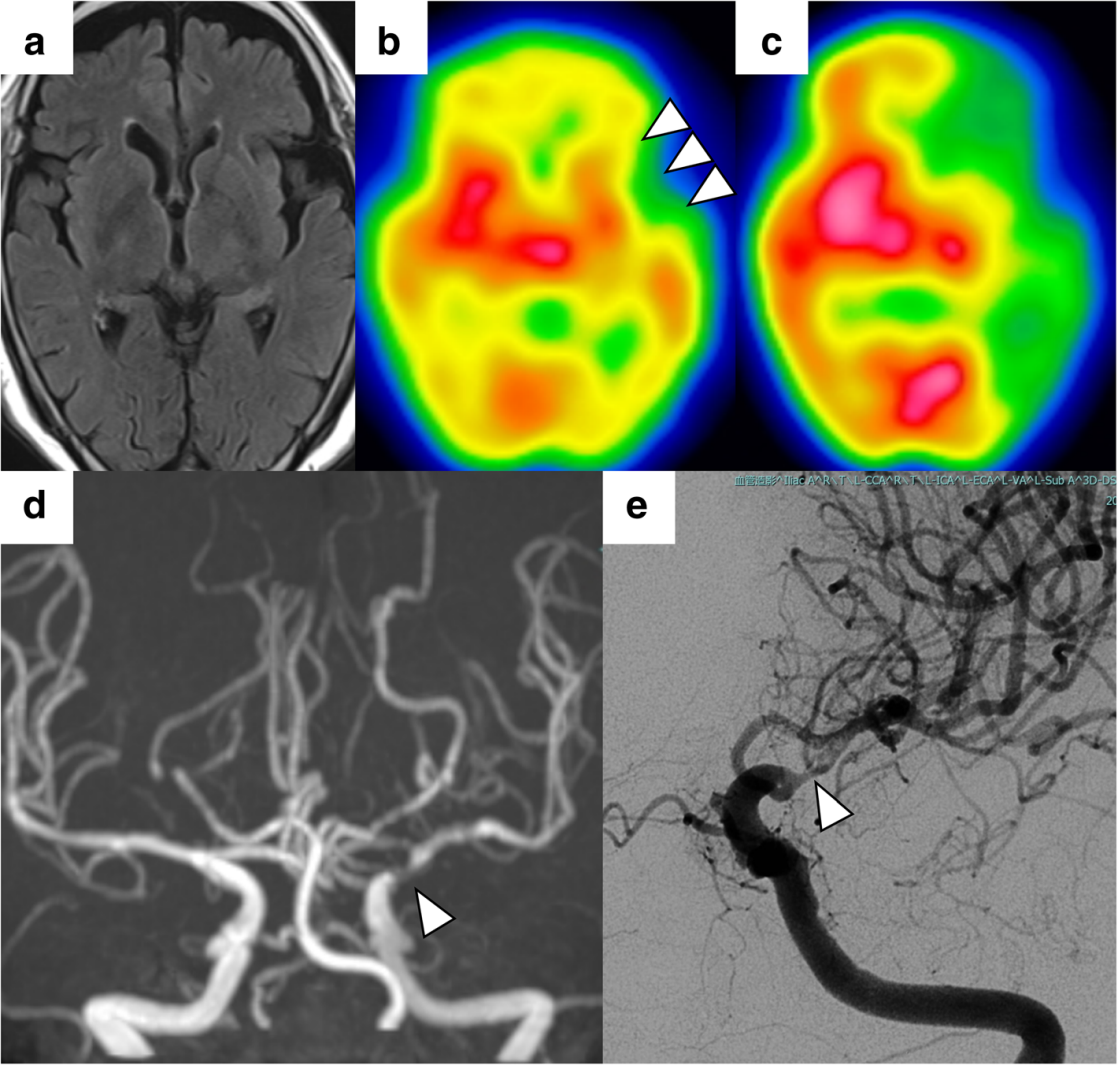
Hemichorea developed in a case of *RNF213*-related vasculopathy showing no ischemic stroke on an MRI fluid-attenuated inversion recovery image (**a**) but reduced cerebral blood flow in a resting state (**b**, arrowheads) and after acetazolamide challenge (**c**) of ^123^I-iodoamphetamine-single photon emission computed tomography with unilateral intracranial stenosis of the left internal carotid artery on MRA (**d**) and digital subtraction angiography (**e**, arrowhead)

**Fig. 2:**
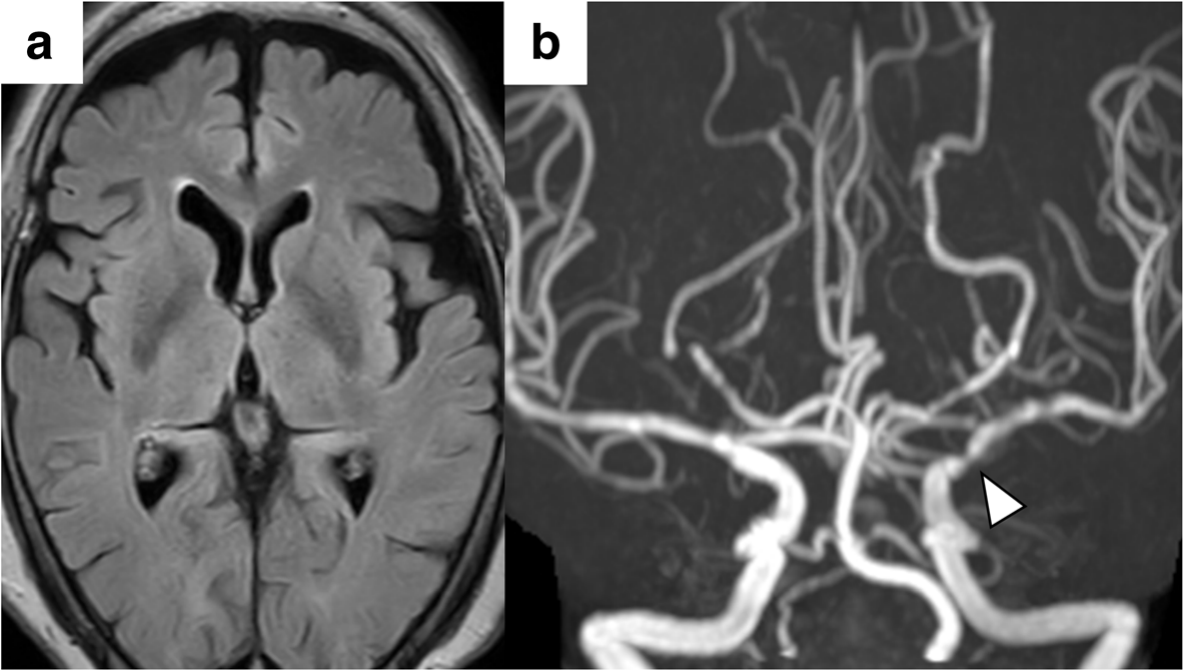
Follow-up head MRI fluid-attenuated inversion recovery image (**a**) and MRA (**b**) taken one year later showed no interval changes, including unilateral intracranial stenosis of the left internal carotid artery (**b**, arrowhead)

An 81-year-old woman noticed choreic movements in the periphery of the right lower extremity at rest, which gradually became more frequent. One month later, she also noticed involuntary movements in the periphery of the right upper extremity and was admitted to our hospital. On admission, she displayed involuntary, irregular, and nonrhythmic movements in the periphery of the right extremities without other neurological deficits. She had a medical history of hypertension and dyslipidemia but no family history of cerebrovascular disorders or chorea. Blood and cerebrospinal fluid tests and cervical and lumbar spine magnetic resonance imaging (MRI) were normal. Head MRI showed no fresh or old cerebral infarction (Fig. 1a). However, ^123^I-iodoamphetamine-single photon emission computed tomography showed cerebral blood flow < 80% in the anterior territory of left middle cerebral artery (MCA) in a resting state (Fig. 1b) and cerebrovascular reactivity < 10% in the broader area supplied by the left MCA after acetazolamide challenge (Fig. 1c). Head magnetic resonance angiography (MRA) and digital subtraction angiography revealed left ICA C1 portion stenosis with poor collateral vessels (Fig. 1d, e). She refused recommended extracranial-intracranial bypass surgery; however, the involuntary movements resolved with haloperidol administration within 3 days, without apparent recurrence from continuation of the medication for a year. Follow-up head MRI and MRA taken 1 year later showed no interval changes (Fig. 2a, b). Genetic testing performed with Taqman probes (TaqMan SNP Genotyping Assays; Applied Biosystems) using a 7300/7500 Real-Time PCR System (Applied Biosystems) [15] revealed the presence of the heterozygous *RNF213* p.R4810K variant.

## Discussion and conclusions

We detail a case of unilateral intracranial ICA stenosis presenting with hemichorea. This case did not meet the diagnostic criteria of MMD [14] because of the presence of unilateral intracranial stenosis of ICA without aberrant vessels in the basal ganglia; however, the patient was found to carry the *RNF213* p.R4810K variant, which has been strongly associated with MMD [16]. We and others have reported this variant as a key factor in ischemic stroke with intracranial arterial stenosis, a common stroke subtype found in East Asian cases, and termed ‘*RNF213*-related vasculopathy’ [13, 16]. This report may indicate a possible explanation of why ICA stenosis leads to chorea more predominantly in East Asia and potentially widens the disease spectrum of *RNF213*-related vasculopathies through inclusion of chorea as an additional clinical symptom. As with MMD, previous reports have suggested a predominance of hemichorea in ICA stenosis in East Asian cases, which we have termed the ‘East-West gradient’ [11], possibly indicating the importance of not only hemodynamic change, but also the ethnicity-specific *RNF213* p.R4810K variant, in hemichorea development. Chorea is thought to be caused by damage to circuitry connecting the basal ganglia with the cerebral cortex [1], as found in cases of MMD with aberrant vessels in the basal ganglia [12]. However, aberrant vessels were not observed in the basal ganglia in the current case, decreasing likelihood of a role in chorea. Alternatively, as *RNF213* encodes a protein containing two ATPases associated with diverse cellular activities and an E3 ligase domain that regulate vascular endothelial function and angiogenesis [17], dysfunctional RNF213 may directly damage neural circuitry, contributing to development of chorea. Indeed, we recently reported endothelial cell specific *RNF213* mutant (human p.R4810K orthologue) transgenic mice had delayed recovery of cerebral blood flow, more profoundly in the basal ganglia than the cerebral cortex, after cerebral hypoperfusion induced by carotid artery stenosis [18]. Therefore, endothelial damage and resultant neural dysfunction in the basal ganglia may be associated with chorea. Additional cases will be required to uncover the mechanism of the genotype-phonotype association, which may be being obscured by the low penetrance of *RNF213* variant and susceptibility to environmental factors [15, 19, 20].

In conclusion, this case may expand and renew the disease concept of *RNF213*-related vasculopathy and elaborate on the pathophysiological mechanisms behind chorea in ICA stenosis.

## Data Availability

Not applicable.

## Abbreviations

ICA: Internal carotid artery
MMD: Moyamoya disease
MCA: Middle cerebral artery
MRI: Magnetic resonance imaging
MRA: Magnetic resonance angiography
